# *LEAFY COTYLEDON1*, a Key Regulator of Seed Development, Is Expressed in Vegetative and Sexual Propagules of *Selaginella moellendorffii*

**DOI:** 10.1371/journal.pone.0067971

**Published:** 2013-06-12

**Authors:** Ryan C. Kirkbride, Robert L. Fischer, John J. Harada

**Affiliations:** 1 Department of Plant Biology, University of California, Davis, California, United States of America, and; Graduate Program in Plant Biology, University of California, Davis, California, United States of America; 2 Department of Plant and Microbial Biology, University of California, Berkeley, California, United States of America; Wuhan University, China

## Abstract

LEAFY COTYLEDON1 (LEC1) is a central regulator of seed development that plays a key role in controlling the maturation phase during which storage macromolecules accumulate and the embryo becomes tolerant of desiccation. We queried the genomes of seedless plants and identified a LEC1 homolog in the lycophyte, *Selaginella* *moellendorffii* , but not in the bryophyte, *Physcomitrella* *patens* . Genetic suppression experiments indicated that *Selaginella LEC1* is the functional ortholog of *Arabidopsis* *LEC1*. Together, these results suggest that *LEC1* originated at least 30 million years before the first seed plants appeared in the fossil record. The accumulation of *Selaginella LEC1* RNA primarily in sexual and asexual reproductive structures suggests its involvement in cellular processes similar to those that occur during the maturation phase of seed development.

## Introduction

The seed is a complex structure that is comprised of three distinct regions: embryo, endosperm, and seed coat. Seed development can be divided conceptually into two temporal phases. During the early morphogenesis phase, the single-celled zygote develops into a multicellular embryo with a shoot-root axis and the embryonic tissue and organ systems [1]. The endosperm develops initially as a syncytium in which nuclei become regionalized to form distinct morphological domains, and it later cellularizes [2]. It is also during this period that ovule integuments differentiate into the seed coat [3]. The maturation phase follows morphogenesis and is characterized by the accumulation of storage macromolecules, such as lipids and proteins, within the embryo and endosperm. The embryo also becomes tolerant of the stresses of desiccation that occur during seed drying [4,5]. At the end of maturation, the embryo and endosperm are quiescent metabolically and arrested developmentally. Under permissive conditions, the mature seed germinates, and growth and differentiation of the seedling begins.

Seed plants constitute the largest and most species-rich group of extant land plants. However, the evolutionary origin of the seed 385 to 365 million years ago (mya) [6] is poorly understood, because early seed plants radiated rapidly and the earliest seed plant lineages are known only from the fossil record [7–9]. Evolution of the seed habit required a number of developmental innovations, including the advent of heterospory, retention and encasement of the megasporangium in maternally-derived integuments to form the ovule, and incorporation of the maturation phase into sporophyte development [10]. The maturation phase represents an intrusive pause in the morphogenesis of the sporophyte that enables the mature embryo to remain quiescent until conditions are favorable for germination [4,5]. By contrast, the life cycle of basal, non-seed bearing land plants is uninterrupted. Despite the importance of the maturation phase, little is known of how it has become integrated into the seed plant life cycle.

LEAFY COTYLEDON1 (LEC1) is a central regulator of seed development with a critical role in regulating the maturation phase (reviewed in 11–13). Loss-of-function mutations in *LEC1* cause defects in storage macromolecule accumulation and the loss of desiccation tolerance, whereas ectopic expression of *LEC1* confers embryonic characteristics to seedlings, including the expression of genes encoding storage macromolecules [14–16]. LEC1 is a novel HAP3 (NF–YB) subunit of the CCAAT-binding (NF–Y) transcription factor [14]. The HAP3 gene family has undergone a dramatic radiation and specialization in land plants as compared with yeast and animals that have single HAP3 genes [17–20]. *Arabidopsis* possesses 13 HAP3 subunits that can be grouped broadly into two classes, the LEC1-type and the non-LEC1-type, based on phylogenetic and functional criteria [19,21]. HAP3 subunits share a highly conserved, central B domain that is flanked by divergent A and C regions. LEC1 and another LEC1-type HAP3 subunit, LEC1-LIKE (L1L), share 16 signature B-domain amino acid residues that differ from residues conserved in non-LEC1-type HAP3 subunits. Moreover, overexpression of LEC1 and L1L confers embryonic characteristics to vegetatively growing plants, whereas ectopic expression of non-LEC1-type HAP3 genes does not induce these traits [19]. Thus, LEC1 represents a functionally specialized HAP3 subunit required for the maturation phase.

Because of the radiation and functional specialization of HAP3 subunits and the central role of LEC1 in seed development, we investigated the origin of LEC1 in basal land plants. We examined two lower plant lineages, *Physcomitrella* *patens* and *Selaginella* *moellendorffii* , representing bryophytes and lycophytes, respectively, to identify the most basal LEC1-type HAP3 subunit and to obtain clues about its role in the evolution of the seed.

## Materials and Methods

Adult *Selaginella* *moellendorffii* plants were purchased (Plant Delights Nursery Inc., Raleigh, NC) or grown from bulbils (generously provided by Jo Ann Banks, Purdue University). Bulbils were surface sterilized (2 min in 70% ethanol, 5 min in 20% bleach, followed by five rinses with sterile water) and grown on one-half strength MS salts agar medium [22] in growth chambers under 56 µmol m-2 s-1, 16 hour light, 8 hour dark at 21°C until they had produced a shoot and root system. At this point, they were transferred to soil and grown in the greenhouse at approximately 26°C, 80% relative humidity, and 57 µmol m-2 s-1 light intensity.

Total RNA was isolated from *S* *. moellendorffii* organs with RNEasy Mini-Kit (Qiagen) and treated twice with DNase (DNA-free kit, Ambion). First-strand cDNA was synthesized with Thermoscript reverse transcriptase (Invitrogen) and used as a template for cloning and qRT-PCR.

DNA sequences for *HAP3* genes were originally identified with BLAST searches against EST databases and trace archives of genomic DNA sequences from the moss, *Physcomitrella* *patens* (http://genome.jgi-psf.org/euk_cur1.html), and the lycophyte, *S* *. moellendorffii* (http://selaginella.genomics.purdue.edu/), to find ancient homologs of *LEC1*. The DNA sequences were confirmed with current genome assemblies (www.phyotozome.net) [23,24]. Coordinates for putative *S* *. moellendorffii* and *P* *. patens* *HAP3* genes are given in Table S1 along with corresponding cDNA accession number. Because the *S* *. mollendorffii* genome contains two nearly identical haplotypes (98.5% identical at the nucleotide level) [23] that have not been organized into chromosome-scale pseudomolecules, we checked ~100 kb of genome sequence flanking HAP3 candidate loci for synteny using BLAST2. This analysis allowed us to identify and remove duplicate loci corresponding to the same chromosome position from the two haplotypes (Figure S1). Only unique *S* *. mollendorffii* loci were retained for analysis. Protein sequences for *Saccharomyces cerevisiae*, *Arabidopsis thaliana*, *S.* mollendorffii and *P* *. patens* HAP3 subunits were aligned using CLUSTALX [25] and trimmed to the B domains. PHYLIP [26] was used to construct maximum parsimony trees with 1000 bootstrap replicates, and the consensus majority rule tree is displayed. The alignment was formatted using BoxShade (http://www.ch.embnet.org/software/BOX_form.html).

*SmLEC1* cDNA was amplified using 5' and 3' SMART-RACE (Clontech). Nested 3' RACE primers were as follows: 5'-CGAGTTCATCAGCTTCATCACCAG-3', 5'-CTCTCGCTCTTCCTCCACAAGTACC-3', 5'-GTACTTGTGGAGGAAGAGCGAGAGC-3' and 5'-CTGGTGATGAAGCTGATGAACTCG-3'. cDNA products were cloned into pCR-2.1 and sequenced.

The *SmLEC1* cDNA clone was inserted into the *LEC1* expression cassette [19]. This construct was transformed into *Agrobacterium tumefaciens* strain GV3101 and transferred into both wild-type Ws-0 and *lec1-1* mutant *Arabidopsis* plants. Suppression of the *lec1* mutation was assessed by the ability of seeds to germinate [14], and genotypes were confirmed by PCR amplification.

Quantitative RT-PCR was done essentially as described previously [27]. Primers for the control RNA, *S* *. moellendorffii* ubiquitin EST (DN839032) were: Sm_Ubi_1F 5'-ATACCATCGGCGATTTGAAG-3',

Sm_Ubi_1R 5'-CGCTTACAAGGAAAGCACCT-3'. Gene-specific primers for *SmLEC1* were: Sm_B_Contig_2F 5'-CAGGACCGCTTCATGCCCAT-3' Sm_B_Contig_2R 5'-GGGTCGGCGTAATCGTCGAA-3'. The statistical significance of mRNA level differences was determined by subjecting all pairwise comparisons of delta-Ct values to Bonferroni-corrected Student’s t-tests [28].

To prepare thin histological sections, bulbils were fixed in triple fixative [29] and dehydrated using a graded water/ethanol series. The samples were then put through a graded ethanol/xylenes series and embedded in paraffin. Bulbils were sectioned at 7 µm on a rotary microtome, mounted on slides, deparaffinized with xylenes and stained with toluidine blue.

Staining for lipid contents was performed using fresh plant material sectioned at ~30 µm using a vibratome. The slides containing sections were then transferred to 50% ethanol for a few seconds and stained with 0.07% Sudan Black B in 70% ethanol for five minutes. Excess stain was removed by immersing slides in 50% ethanol for 1 minute, followed by mounting in glycerol and imaging.

## Results

**Figure 1:**
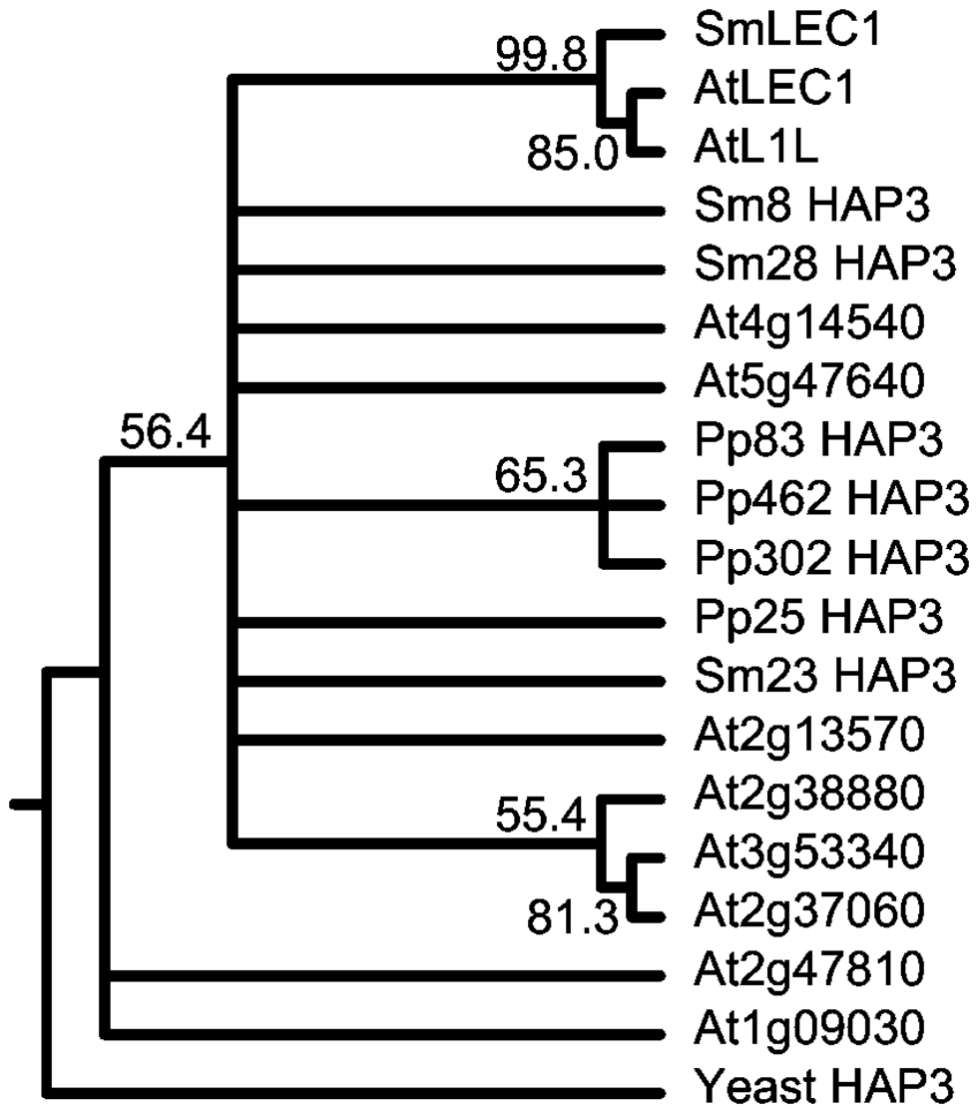
Phylogenetic tree based on the B domains of HAP3 subunits from yeast, *Arabidopsis* , *S.* moellendorffii, and *P* *. patens* . Nodes with bootstrap values below 50 were collapsed.

We identified several DNA sequences corresponding to the B domain of HAP3 subunits in both *S* *. moellendorffii* and *P* *. patens* (see Materials and Methods). As shown in Figure 1, phylogenetic analysis of these HAP3 B domain amino acid sequences from *Arabidopsis* and *S. cerevisiae* suggested that *S* *. moellendorffii* and *P* *. patens* possessed non-LEC1-type HAP3 subunits, although much of the phylogeny of the non-LEC1-type HAP3 subunits was not fully resolved. By contrast, a LEC1-type HAP3 subunit that grouped in a well-supported, monophyletic clade with *Arabidopsis* LEC1 and L1L was detected only *S* *. moellendorffii* and not *P* *. patens* . Thus, a LEC1-type HAP3 subunit was detected in *S* *. moellendorffii* but not in *P* *. patens* .

**Figure 2:**
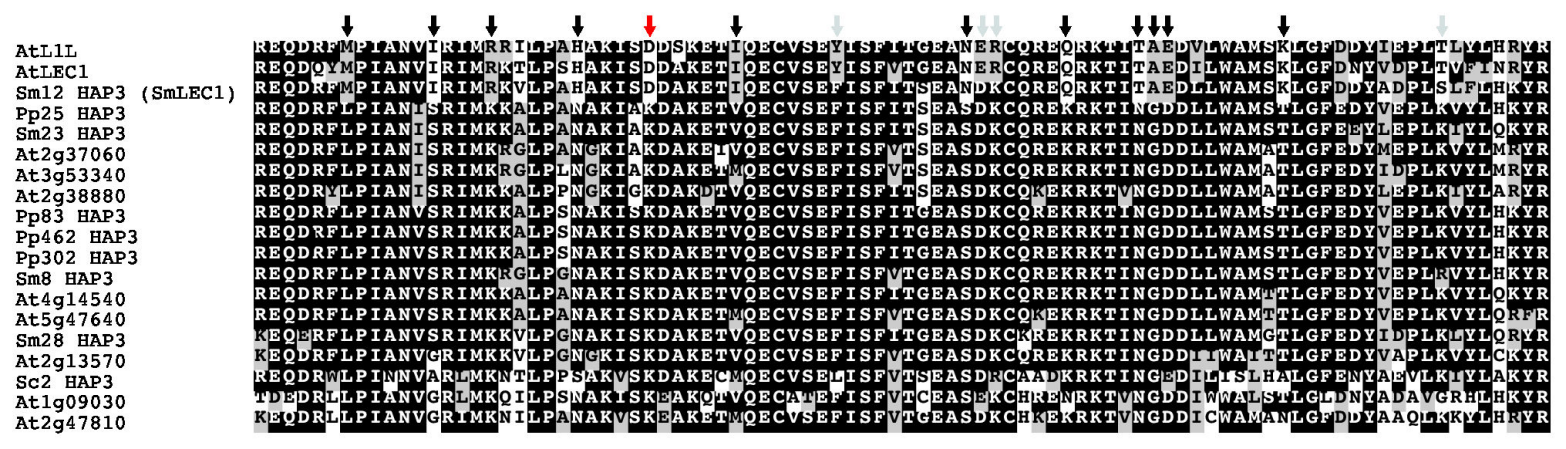
Amino acid sequence alignment of the B domains of yeast, *Arabidopsis* and putative *S* *. moellendorffii* and *P* *. patens* HAP3 subunits. Positions of residues diagnostic for the LEC1-type B domain are marked with arrows. Red arrow indicates the key Asp residue required for LEC1 activity. Grey and black arrows, respectively, show residues that are different or identical between *S* *. moellendorffii* and *Arabidopsis* LEC1-type HAP3 subunits. Amino acids identical to the consensus (>50%) sequence are shown with a black background, whereas similar amino acids are shown with a grey background.

A single gene, designated *SmLEC1*, encoding the *S* *. moellendorffii* LEC1-type HAP3 subunit was identified. We showed that the gene was expressed by cloning its corresponding cDNA. The putative SmLEC1 polypeptide consists of 175 amino acids, with a 90-residue B domain that shares 83% amino acid identity with the *Arabidopsis* LEC1 B domain (Figure 2). More significantly, 12 of the 16 signature amino acid residues characteristic of angiosperm LEC1-type B domains [19] were conserved in SmLEC1, including the essential Asp residue at position 28 that is required for LEC1 function [21]. The A and C regions of SmLEC1, flanking the LEC1-type B domain, were not conserved between *S* *. moellendorffii* and *Arabidopsis* , consistent with the reports that *Arabidopsis* LEC1 and L1L do not share similarity in A and C regions [19] and that the B domain is responsible for specifying LEC1-type function [21].

To determine if SmLEC1 and *Arabidopsis* LEC1 have orthologous functions, we conducted genetic suppression assays using the *Arabidopsis* *lec1-1* mutant. *lec1-1* mutant embryos are intolerant of seed desiccation, and mutant seeds do not germinate after drying [16,30]. The ability of transgenic mutant seeds to germinate is a indication that the transgene can genetically suppress the *lec1* mutation [14]. We fused the *SmLEC1* cDNA clone with *Arabidopsis* DNA sequences 5' and 3' of the LEC1 protein coding region [19] and transferred the transgene into *lec1-1 mutants*. *lec1-1* mutant plants transformed with *SmLEC1*, *Arabidopsis* *LEC1*, and an empty vector control vector generated 0.73% (51 of 7,015), 0.40% (56 of 13,950), and 0.062% (5 of 8,064) viable seedlings, respectively. The germination percentages reflected both the transformation efficiency and ability of the transgenes to suppress the *lec1* mutation. This result suggests strongly that *SmLEC1* is orthologous and functionally conserved with *Arabidopsis* *LEC1*.

**Figure 3:**
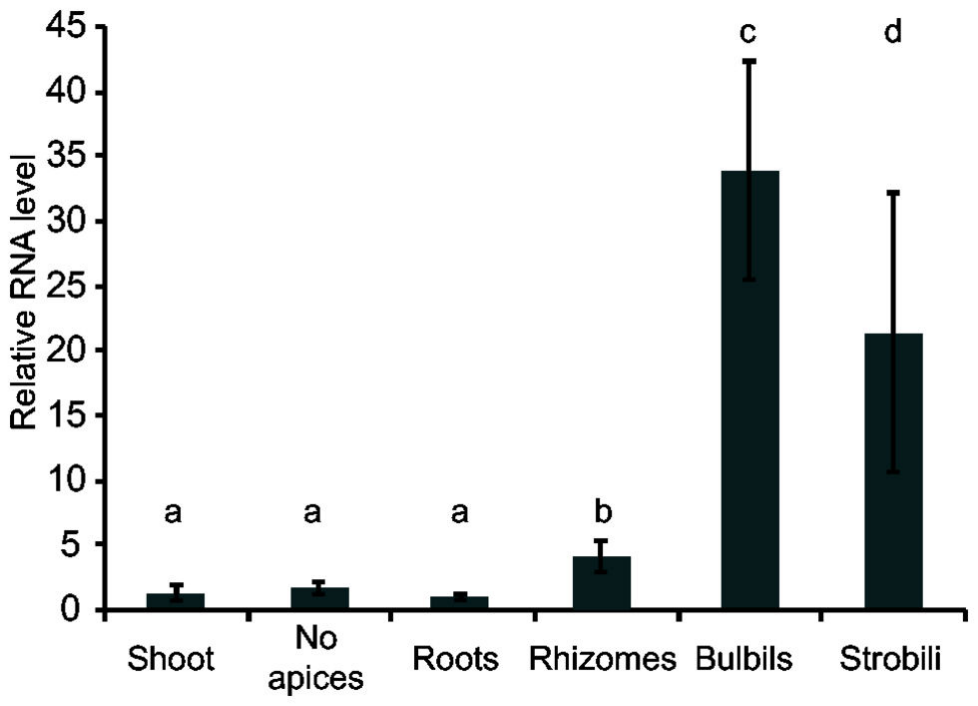
*SmLEC1* is expressed in vegetative and reproductive propagules. qRT-PCR survey of *SmLEC1* mRNA levels in various *S* *. moellendorffii* organs. No apices are shoot samples with growing tip removed to avoid sampling incipient propagules. Relative quantification of RNA levels was calculated using ubiquitin as a control. Error bars indicate standard deviations. The following comparisons were determined to be significantly different (p < 0.05): a vs c, b vs c, and a vs d.

**Figure 4:**
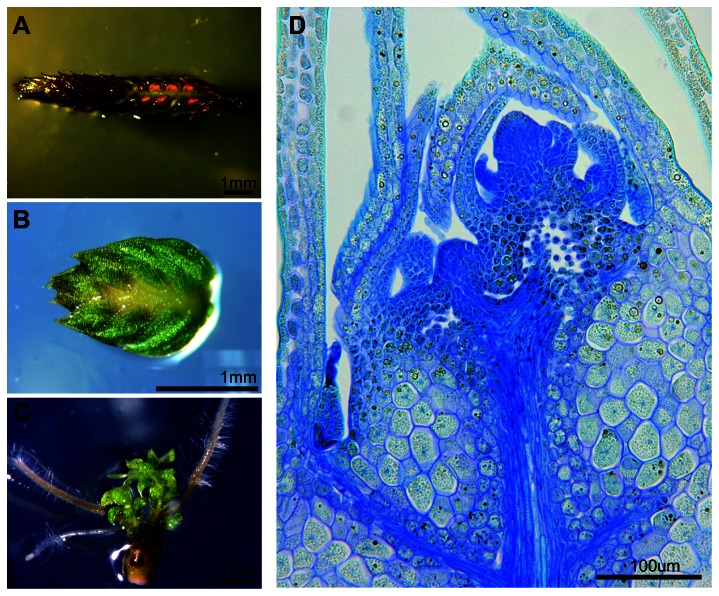
Vegetative and reproductive propagules of *S* *. moellendorffii* . (A) Strobilus with basal microphylls removed to reveal axillary sporangia. (B) Mature, dormant bulbil. (C) Mature bulbil showing new shoot and root growth from apical and angle meristems respectively. (D) Longitudinal section of dormant bulbil stained with toluidine blue. showing the shoot apex and enlarged cells in the main body of the bulbil.

**Figure 5:**
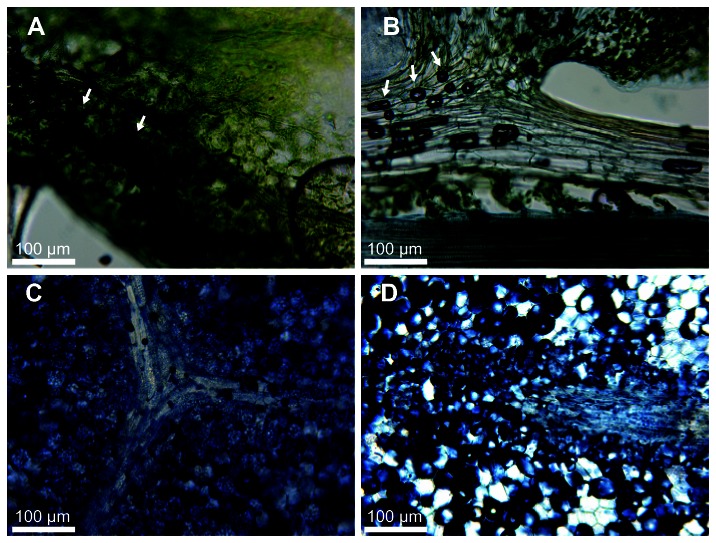
Lipid storage in bulbils. Vibratome-cut sections were stained with Sudan Black B to identify cells with neutral lipids. (A) Section through an *Arabidopsis* leaf with a low storage lipid content. (B) Stem and leaf section from *S* *. moellendorffii* . (C) Section through a pumpkin oilseed with high storage lipid content. (D) *S* *. moellendorffii* bulbil section. Stained lipids appears as dark blue or black. Air bubbles in A and B (arrows) appear dark and do not indicate staining. The images are representative of material from at least three different plants.

To obtain insight into the role of LEC1 in a basal land plant, we analyzed *SmLEC1* RNA levels in *S* *. moellendorffii* shoots, shoots with apices removed to avoid incipient bulbils, rhizomes, roots, bulbils and strobili. Figure 3 shows that *SmLEC1 RNA* was present at highest levels in strobili and bulbils. Strobili, as shown in Figure 4A, contain both the female and male reproductive organs, megasporangia and microsporangia, that are located in the axils of microphylls, although microsporangia are far more abundant [31]. Bulbils are a loose class of vegetative propagules that are produced from various organs. *S* *. moellendorffii* bulbils are enlarged shoot apices that have ceased elongation and microphyll production (Figure 4B). They are quiescent developmentally and can be dispersed after being detached from the plant, retaining viability for approximately one year. Under appropriate conditions, bulbils can regenerate the adult plant through reactivation of shoot growth at the bulbil tip along with root formation from angle meristems (Figure 4C) [32]. Figure 4D shows that bulbils possessed enlarged cells reminiscent of those containing storage macromolecules. Consistent with this interpretation, we showed that sections through bulbils stained strongly for lipids (Figure 5D) similar to those obtained from an oil-storing angiosperm seed (Figure 5C). By contrast, sections from stems and leaves of *S* *. moellendorffii* and angiosperm leaves (Figure 5B and 5A, respectively) did not stain substantially for lipids. We conclude that *S* *. moellendorffii* bulbils expressed LEC1-type genes and accumulated lipids, similar to what occurs in seeds.

## Discussion

Our results demonstrate that a gene encoding a functional LEC1-type HAP3 subunit is present in a lycophyte but is not detected in a bryophyte. These findings place the origin of LEC1 at approximately 400 to 430 mya [33] which is approximately 20 to 30 million years before the appearance of the first seed plants. Although the sequence of the *P* *. patens* genome is largely complete [24], we cannot formally exclude the possibility that the *LEC1* gene is present in an unsequenced region of the *P* *. patens* genome. It is also possible that a *LEC1*-type gene is present in other bryophytes and its absence in *P* *. patens* is due to a lineage-specific loss. Similar results have been reported by Xie et al. [34] who identified *LEC1-type HAP3* genes in other lycophyte and fern genomes. The mode of origin of the LEC1-type B domain, however, remains uncertain. The sudden appearance of the LEC1-type gene in evolutionary time and a lack of evidence of recent duplication among other HAP3 genes make it unlikely that the appearance of LEC1 was the result of genome duplication. Others have postulated that the absence of introns in *SmLEC1* relative to another class of basal land plant HAP3 genes may support an origin via a retrotransposition event [34].

Because LEC1-type HAP3 genes appeared prior to the origin of seed plants, we were interested to learn their physiological roles in *Sellaginella* and whether these roles relate to LEC1 function in seed development. In higher plants, LEC1 is a major regulator of the maturation phase, a developmental innovation critical for the seed habit [15,16]. For example, overexpression of *LEC1* causes the induction of genes encoding storage proteins and fatty acid biosynthetic enzymes and the accumulation of fatty acids [14,35,36]. Features of the maturation phase that are critical for the seed habit include the acquisition of desiccation tolerance that permits the embryo to withstand the stresses of drying and become metabolically quiescent and the accumulation of macromolecular reserves, such as storage proteins and lipids that are used as a nutrient source by the developing embryo and/or seedling. Thus, integration of the maturation phase into the sporophytic generation enables a period of developmental and metabolic quiescence in which the embryo remains arrested until conditions are appropriate for subsequent vegetative and reproductive growth [4,5].

Lycophytes are basal land plants that represent the first extant group that diverged after the lineages that make up the bryophyte grade. They are the earliest diverging vascular plant lineage possessing a dominant sporophytic generation and true vasculature. Although some lycophyte genera, including *Selaginella* , are heterosporous, these instances of heterospory were derived independently from that of seed plants [37]. Because lycophytes are seedless plants that have not integrated the maturation phase into their life cycles, the *LEC1*-type gene must have evolved in lycophytes to fulfill other physiological processes.

Two sets of physiological processes that characterize lycophytes and some other basal land plants overlap with those that occur in maturation-phase seeds. First, several structures, such as the bulbils of lycophytes and monilophytes and gemmae of bryophytes, serve as specialized propagules that accumulate storage macromolecules [38,39]. For example, we showed that *S* *. molenlendorffii* bulbils possess substantial quantities of lipid (Figure 5). Second, lycophytes acquire the ability to withstand desiccation at specialized developmental stages or environmental conditions. Spores, particularly the megaspores of heterosporous plants such as *S* *. moellendorffii* , undergo desiccation prior to giving rise to viable gametophytes [40]. Furthermore, gametophytes and sporophytes of several lower plants, including certain species of *Selaginella* known as resurrection plants, desiccate and become quiescent upon drying, yet they remain viable [41].

Based on the finding that *SmLEC1* RNA is present at high levels in strobili and bulbils and at low levels elsewhere in the plant (Figure 3), we hypothesize that *SmLEC1* may function to induce storage lipid accumulation in *S* *. mollendorffi* bulbils and confer desiccation tolerance to megaspores. The findings that ectopic expression of *Arabidopsis* *LEC1* induces storage lipid accumulation in vegetative *Arabidopsis* organs [35] and that *LEC1-type HAP3* RNA levels are upregulated in desiccating foliage leaves of the resurrection plant, *Selaginella* *sinensis* [34] are consistent with this hypothesis. A role for *SmLEC1* in the accumulation of storage reserves and the acquisition of desiccation tolerance would suggest that LEC1 was coopted in angiosperms for its role in seed development. Alternatively, because RNA encoding a LEC1-type HAP3 subunit is detected in *S* *. mollendorffii* strobili and pine pollen cones [19], LEC1-type HAP3 subunits may have evolved with an original role in gametophyte development. While functional characterization of SmLEC1 in *S* *. moellendorffii* remains difficult in the absence of a gene transfer system, further exploration of the role of the LEC1-type HAP3 subunits in other basal land plants should provide insight into ancient processes regulated by LEC1 that were coopted for seed development.

## Supporting Information

Figure S1 **Identification of *S. moellendorffii* *HAP3* haplotypes.**
The sequenced *S* *. moellendorffii* genome contains two haplotypes. Approximately 100 kb regions surrounding HAP3 alleles were compared to identify haplotypes. Dot Matrix View of BLAST2 alignments show exceptionally high sequence alignment (slanted lines) of HAP3 alleles, indicating that the pairs occur in nearly identical scaffolds and not likely to be distinct loci.
(EPS)

Table S1 **Genomic locations of all putative genes encoding HAP3 subunits identified in this study.**
Scaffold and coordinate identifiers correspond to the *Physcomitrella* *patens* 1.6 genome release and the *Selaginella* *moellendorffii* 1.0 genome release.
(DOCX)
