# Supplementary material for: LEAFY COTYLEDON1, a Key Regulator of Seed Development, Is Expressed in Vegetative and Sexual Propagules of Selaginella moellendorffii
Source: PLoS One. 2013 Jun 12;8(6):e67971. doi: 10.1371/journal.pone.0067971 (PMC3680378; doi:10.1371/journal.pone.0067971)
Supplement: Table S1 — Genomic locations of all putative genes encoding HAP3 subunits identified in this study. Scaffold and coordinate identifiers correspond to the Physcomitrella patens 1.6 genome release and the Selaginella moellendorffii 1.0 genome release. (DOCX) [file pone.0067971.s002.docx]

Table S1. Genomic locations of all putative genes encoding HAP3 subunits identified in this study

| Species | Genomic Scaffold | Start Coordinates | Stop Coordinates | Supporting cDNA Genbank ID |
| --- | --- | --- | --- | --- |
| *P. patens* | 25 | 159844 | 160832 | BJ947478 |
| *P. patens* | 302 | 242861 | 243531 | BJ947478 |
| *P. patens* | 83 | 1247078 | 1247816 | BJ941461 |
| *P. patens* | 462 | 48306 | 48716 | BJ941461 |
| *S. moellendorffii* | 76 | 183155 | 183424 | This study |
| *S. moellendorffii* | 12 | 1056303 | 1056572 | This study |
| *S. moellendorffii* | 28 | 1490308 | 1490577 | FE517628.1 |
| *S. moellendorffii* | 22 | 1200878 | 1201147 | FE517628.1 |
| *S. moellendorffii* | 8 | 948331 | 948600 | FE474037.1 |
| *S. moellendorffii* | 0 | 4491704 | 4491973 | FE474037.1 |
| *S. moellendorffii* | 23 | 333595 | 333926 | FE474595.1 |
| *S. moellendorffii* | 58 | 630593 | 630918 | FE474595.1 |
|  |  |  |  |  |
